# Antibiotic Combinations with Daptomycin for Treatment of *Staphylococcus aureus* Infections

**DOI:** 10.1155/2011/619321

**Published:** 2011-04-18

**Authors:** Kristina Nadrah, Franc Strle

**Affiliations:** Department of Infectious Diseases, University Medical Centre Ljubljana, Japljeva 2, 1525 Ljubljana, Slovenia

## Abstract

Daptomycin is a lipopeptide antibiotic with a unique mechanism of action on Gram-positive bacteria. It is approved for treatment of skin and soft-tissue infections with Gram-positive bacteria, bacteraemia and right-sided infective endocarditis caused by *Staphylococcus aureus*. Diminishing susceptibility of *S. aureus* to daptomycin during treatment of complicated infections and clinical failure have been described. Combinations of daptomycin with other antibiotics including gentamicin, rifampin, beta-lactams, trimethoprim/sulfamethoxazole (TMP-SMX), or clarithromycin present a new approach for therapy. *In vitro* and animal studies have shown that such combinations may, in some cases, be superior to daptomycin monotherapy. In this paper we focus on the antibiotic combinations for complicated *S. aureus* infections.

## 1. Introduction

Daptomycin, an antibiotic with a new mechanism of action and low occurrence of spontaneous resistance, came to market in 2003 in the USA and in 2006 in Europe. In spite of its many promises for treatment of infections caused by Gram-positive bacteria, reports on clinical failure and diminished* in vitro* susceptibility soon came forward. Alongside, it became clear that daptomycin monotherapy of biofilm-related and deep-seated infections is often not effective. Due to limited clinical settings in which daptomycin is effective, diminishing susceptibility to daptomycin, emerging resistance to linezolid, and slow development of new antibiotics, clinicians have difficulties treating serious *Staphylococcus aureus* infections. Therefore, combinations of daptomycin with other antibiotics are extensively studied as a potential new therapeutic strategy. In this review, we will focus on the antibiotic combinations for complicated *S. aureus *infections.

## 2. Daptomycin Pharmacology

Daptomycin is a novel lipopeptide antibiotic approved for treatment of complicated skin and soft-tissue infections caused by Gram-positive bacteria, and for *S. aureus* bacteraemia and right-sided endocarditis [1]. It is not active in low respiratory tract infections, because lung surfactant forms complexes with daptomycin thereby inactivating it [2]. 

Daptomycin mechanism of action is complex and not entirely understood. Research so far indicates that it acts on cell membrane [3, 4] and also inhibits the synthesis of lipoteichoic acid, necessary for cell wall synthesis [4]. 

Daptomycin structure consists of an anionic core and a lipophilic tail. By binding divalent calcium and forming Ca^2+^-complexes, the net charge of the molecule becomes positive and thus enables electrostatic interactions with negatively charged cell membrane. It then binds to cell membrane, where it forms oligomers and subsequently channels through which intracellular ions, like potassium, leak out of the cell, diminishing cell membrane negative potential and causing cell death [3]. This initial model was later modified according to the observation that daptomycin forms micelle-like structures in the medium and binds to the membrane in the already oligomerized form [5]. However, Hobbs et al. found that changes in dissipation of membrane potential and leakage of intracellular material occur rather late, after cell death, and with no significant alterations in membrane integrity [6]. Similarly, no significant cell lysis was found by electron microscopy and the membrane integrity probes used on *S. aureus* treated *in vitro* with daptomycin [7]. In addition, analogous to cell wall active antibiotics, daptomycin was found to upregulate cell wall stress stimulon genes [8]. The gene expression profile of *S. aureus* after being exposed to daptomycin is similar to profiles developed after exposure to cell wall active antibiotics, such as beta-lactams, as well as to compounds which disrupt cell membrane, like carbonyl cyanide *m*-chlorophenylhydrazone [8]. Thus, beside acting on the cell membrane, daptomycin likely acts on several other targets.

Daptomycin activity is sensitive to the inoculum of the infectious micro-organism [9]. This is probably due to the decrease of the local effective antibiotic concentration at high inoculum [10]. Therefore, sufficient serum concentrations are essential for clearance of bacteria from the infection site. 

The current susceptibility cutoff MIC of* S. aureus *isolates to daptomycin is set at 1 mg/L [11]. In some cases, susceptibility profile is heterogeneous [12]. No MIC creep like in the case of vancomycin has been noticed [13–15], even in the isolates collected over several decades [11]. Since unequivocal criteria for resistance have not been defined yet, the term non-susceptibility is used instead of resistance.

## 3. Mechanisms of Non-susceptibility to Daptomycin

Strains of* S. aureus *non-susceptible to daptomycin have been obtained from clinical cases, by *in vitro* selection, and by chemical mutagenesis. Frequency of spontaneous daptomycin resistance in* S. aureus *is low (10^−10^) [12]. Serial passages in subinhibitory concentrations of daptomycin give rises of MICs by a factor of 8–32; the same is valid for chemical mutagenesis [12, 16, 17]. 

Several genes have been implicated in* S. aureus *non-susceptibility to daptomycin. Overexpression and mutations in *mpr*F [18–20] which encodes lysyl-phosphatidylglycerol (LPG) synthetase and flippase, and *yyc*G which encodes histidine kinase in a two-component sensor regulatory system of YycF/YycG [16, 20] were identified in clinical isolates and laboratory-derived strains, while *rpo*B [20, 21] and *rpo*C [16] which encode *β* and *β* ^'^ subunits of RNA polymerase were detected only in *in vitro* selected laboratory non-susceptible strains. *In vitro* insertional mutation of *csp*B [22], a cold shock gene, led to increased susceptibility to daptomycin in a daptomycin non-susceptible strain of *S. aureus*. Several mutants obtained *in vitro* and some of the clinical isolates contain at least one of these genetic changes; a combination of the genetic alterations seems to have an additive effect on the MIC value [20]. However, some daptomycin non-susceptible strains do not have any of these alterations [23, 24], indicating that the mechanism of “resistance” is probably multifactorial. 

The phenotypic alterations in non-susceptible* S. aureus *can be grouped into (1) changes in cell wall structure and turnover; (2) changes in membrane composition, membrane structure, and membrane potential; (3) modifications in sensitivity to depolarization, autolysis, and permeabilization. In some cases, thicker cell wall had been correlated to non-susceptibility [18, 20, 21, 25], but this was not a uniform finding; for example, Yang et al. found no changes in cell wall thickness in a clinical meticillin-resistant *S. aureus* (MRSA) isolate with diminished susceptibility to daptomycin which developed during treatment [19].* S. aureus *strains non-susceptible to daptomycin had an increased synthesis of LPG and increased translocation of LPG to the outer membrane, and hence modifications in membrane fluidity and electrostatic potential [19, 20, 26]. A loss of an unidentified 81 kD membrane protein has also been described in a non-susceptible clinical isolate [17]. 

So far, most cases of clinically acquired non-susceptibility of* S. aureus *to daptomycin occurred in a setting of inadequate dosing [17] and/or deep-seated, high-inoculum, and biofilm-related infective, such as infectious endocarditis (IE) [17, 19, 26, 27] or bone infections [24]. In these cases, the effective concentration of daptomycin at the site of the highest bacterial density is low [10], and activity is further diminished by the stationary phase of bacteria in biofilm which has been associated with such infections [28].

## 4. Relationship between Diminished Susceptibility to Vancomycin and Daptomycin

Diminished susceptibility of* S. aureus *to vancomycin has been associated with a development of diminished susceptibility to daptomycin. This has been established for multidrug resistant (MDR)* S. aureus*, for MRSA, as well as for meticillin-susceptible *S. aureus* (MSSA). Daptomycin is often prescribed for treatment of MDR* S. aureus *infections if vancomycin therapy fails. Clinical experience indicates that strains of* S. aureus *with diminished susceptibility to vancomycin develop a diminished susceptibility to daptomycin during treatment [27, 29–31] or even in absence of daptomycin exposure [32, 33]. Several studies found that diminished susceptibility to vancomycin in MRSA is associated with diminished susceptibility to daptomycin [32], and, according to some reports, that it correlates well with increased cell wall thickness [25, 27]. The highest MICs of vancomycin intermediate *S. aureus* (VISA)/MRSA, observed in deep-seated infections such as septic arthritis and osteomyelitis, are often associated with higher MICs for daptomycin or even with non-susceptibility to daptomycin [33]. In the case of MRSA IE and septic thrombophlebitis, the patient's strain developed non-susceptibility to glycopeptides during treatment with glycopeptides, and, after switching therapy to daptomycin, a non-susceptibility to daptomycin. MRSA was successfully eradicated from the bloodstream only after therapy with linezolid and fusidic acid [27]. A similar relationship was found for MSSA: diminished susceptibility to vancomycin which developed during therapy of patients with osteomyelitis [34] or IE [26] was associated with a higher MIC to daptomycin even though the patients had never received it. Subsequent analysis found that vancomycin and daptomycin exhibited a reduced bactericidal activity towards the non-susceptible examined strain and a lesser degree of its chemical autolysis was observed [34]. Nevertheless, *in vitro* time-kill studies show that daptomycin retains bactericidal activity in spite of increased MIC provided that the concentrations exceed MIC by several times [31] (Table 1). 

However, not all studies confirmed that diminished susceptibility of *S. aureus* to vancomycin is associated with diminished susceptibility to daptomycin. In a retrospective study of the influence of previous vancomycin therapy on MIC and bactericidal activity of daptomycin and vancomycin in *S. aureus* (isolates from patients who received vancomycin within 30 days prior to development of MRSA bacteraemia were compared with isolates from those who did not receive the drug), higher MICs and decreased killing was established only for vancomycin but not for daptomycin [35].

## 5. Antibiotic Combinations with Daptomycin

In some groups of patients, such as those infected with *S. aureus *strains which have diminished susceptibility to both vancomycin and daptomycin, especially in the case of MDR *S. aureus*, or in patients with deep-seated daptomycin susceptible *S. aureus* infections, monotherapy with daptomycin at approved doses (4–6 mg/kg/day) is frequently not effective and may even yield non-susceptible isolates. In such patients, treatment options are highly limited, and addition of another antibiotic to daptomycin may be beneficial. The combinations can be divided into three groups: (1) classical combinations of daptomycin and gentamicin or rifampicin; (2) combinations of daptomycin and beta-lactams; (3) combinations of daptomycin with bacteriostatic antibiotics such as trimethoprim/sulfamethoxazole (TMP-SMX) or clarithromycin. Findings on *in vivo* and *in vitro* synergy of antibiotic combinations are depicted on Tables 2 and 3.

### 5.1. Combinations of Daptomycin with Gentamicin or Rifampin

#### 5.1.1. Infective Endocarditis

Standard treatment of left-sided IE caused by* S. aureus *usually includes two antibiotics with a synergistic bactericidal action, typically a cell wall synthesis inhibitor and an aminoglycoside, with the addition of rifampicin in case of infected prosthetic material [36]. Study on a small number of severely ill patients with left-sided *S. aureus* endocarditis revealed that daptomycin monotherapy was inferior to standard therapy, and that both treatment groups had a very low success rate (11% and 22%, resp.) [1]. A retrospective analysis of data from daptomycin outcomes registry [37, 38] suggested that the overall success of treatment of the left-sided IE caused by* S. aureus *may be as high as 60% with the majority of the patients receiving daptomycin in combination with other antibiotics. These findings were a stimulus for the assessment of the efficacy of daptomycin in combination with other antibiotics, initially with gentamicin or rifampicin, that is, antibiotics used for standard treatment. 

Combination of daptomycin with gentamicin or rifampicin was tested *in vitro* as well as in *in vivo* animal models of IE [39]. *E*test with MSSA/heterogeneous glycopeptide-intermediate *S. aureus* (hGISA) and MRSA/GISA showed synergy of daptomycin-gentamicin combination for both types of strains used while the time-kill studies confirmed the synergy only in the case of MSSA/hGISA [40]. Combinations of daptomycin and rifampicin were indifferent in both assays [40]. In an *in vitro* pharmacodynamic model of IE with simulated endocardial vegetations and daptomycin susceptible MRSA strain, the addition of rifampicin and gentamicin substantially delayed or even antagonized the bactericidal effect of daptomycin [39]. Similar results were found in an *in vivo* rabbit model of aortic IE caused by daptomycin-susceptible MRSA: not only was the addition of gentamicin or rifampicin to daptomycin at 6 mg/kg/day of no beneficial value, the combination treatment was less successful in lowering the bacterial cell counts than daptomycin monotherapy [41]. 

However, in a clinical case of a mitral valve endocarditis with heterogeneous population of *S. aureus*, where one population was fully susceptible to vancomycin and daptomycin and the other non-susceptible, the addition of rifampicin to a high dose of daptomycin (12 mg/kg/day) resulted in treatment success with sterile blood cultures in 72 hours [42].

#### 5.1.2. Bone Infections, Foreign-Body Infections, and Biofilms

In general, antibiotic combinations with rifampicin more successfully treat prosthetic devices-related staphylococcal infections [43, 44] and more efficiently eradicate *S. aureus* biofilm than monotherapy without rifampicin [45]. Because penetration of antistaphylococcal antibiotics into the biofilm is variable [46] and the bacteria are in a senescent state [28], serum concentrations of the anti-staphylococcal drug may play a role in successful eradication of staphylococci from a biofilm [46]. In addition, activity of several antibiotics, including daptomycin, is concentration dependent, and thus enhanced with higher serum concentrations [4]. Penetration of rifampicin [47] and daptomycin [48] in an *S. epidermidis* biofilm is good, but, in the absence of data, we may only speculate that the same is valid for *S. aureus* biofilms. 

Information on the efficacy of daptomycin alone and in combination with rifampicin for treatment of *S. aureus* bone and foreign-body infections is rather incomplete, and limited to *in vitro* testing and *in vivo* animal model investigations; the findings of the studies are not completely unequivocal. An *in vivo* study using a rat model of foreign-body infection with a fully susceptible MSSA has found that daptomycin monotherapy at low doses (corresponding to human dose of 4 mg/kg/day) is as effective as vancomycin but can lead to diminished susceptibility to daptomycin which develops during treatment [49]. In an animal model of acute osteomyelitis caused by MRSA susceptible to vancomycin, daptomycin, and rifampicin, monotherapy with daptomycin (6 mg/kg/day) was not successful and in some cases diminished susceptibility occurred during therapy. Addition of rifampicin resulted in a more frequent eradication of bacteria from the bone and prevented the emergence of resistant strains [50]. Similarly, in a guinea pig foreign-body infection model with MRSA susceptible to daptomycin, the addition of rifampicin to an intermediate dose of daptomycin (6 mg/kg/day) resulted in treatment success in two thirds of the animals whereas monotherapy was ineffective [51]. Higher doses of daptomycin seem to be better in eradicating the bacteria, but combinations with rifampicin are even more efficient. A comparison of daptomycin (corresponding to human dose of 10 mg/kg/day) and rifampicin monotherapy in a rat model of foreign-body infection with MRSA susceptible to daptomycin and rifampicin has shown that both therapies are equally effective, but rifampicin resistance developed during treatment in 60% [52]. In a rat model of foreign-body infection with MRSA susceptible to vancomycin, daptomycin, and rifampicin, monotherapy with a high dose of daptomycin (10 mg/kg/day) effectively decreased bacterial cell counts (over 3 log CFU/ml by day 11); the combination of high-dose daptomycin and rifampicin was even more successful (a decrease in bacterial cell counts over 4.5 log CFU/mL by day 11) and cured 94% of the animals whereas the standard dose of daptomycin (6 mg/kg/day) combined with rifampicin cured only two-thirds although being similarly bactericidal when compared to the high-dose daptomycin combined with rifampicin* in vitro* [53]. However, in an *in vitro* model of biofilm with MRSA susceptible to daptomycin, monotherapy with daptomycin at a high dose (10 mg/kg/day) was only minimally effective in diminishing the biofilm cell counts within 72 h whereas the addition of rifampicin enhanced the bactericidal activity of daptomycin against bacteria in biofilm [54]. 

The reason for the discrepancy in rifampicin action among the results obtained in animal models of IE and foreign-body infection is not clear. Recently, Olson et al. proposed that rifampicin exerts the main bactericidal action on a biofilm while anti-staphylococcal antibiotics in the combination with rifampicin only prevent the outgrowth of rifampicin-resistant mutants which are known to emerge frequently and in high numbers during monotherapy with rifampicin in a laboratory setting [55].

### 5.2. Combinations of Daptomycin and Beta-Lactams

Combinations of daptomycin with beta-lactams have a basis in an observation that in some cases MRSA, which develops a diminished susceptibility to vancomycin or daptomycin, becomes more susceptible to oxacillin, a so-called “see-saw” effect [56]. An example of such pathogen is MDR* S. aureus *which becomes non-susceptible to daptomycin. 

Various combinations have been tested in *in vitro* and animal models, most commonly daptomycin combined with a beta-lactam at a fraction of its MIC. 

In *in vitro* time-kill experiments with MRSA susceptible to daptomycin, synergy was found between daptomycin at 0.5 MIC and 32 mg/L oxacillin (oxacillin MIC for the majority of tested strains ≥256 mg/l). Aminopenicillins, such as ampicillin, showed synergy at even lower concentrations (2–8 mg/l ampicillin in a combination with ampicillin/sulbactam) [57]. 

Similarly, addition of subtherapeutic levels of beta-lactams to daptomycin delayed or prevented the emergence of diminished susceptibility to daptomycin in *in vitro* selection studies performed on four homogenously daptomycin susceptible MRSA which were also vancomycin-susceptible (VSSA) and two heterogeneously daptomycin susceptible and methicillin-resistant GISA strains. The MICs increased 2–4 times over the baseline in case of MRSA and only two-fold in case of GISA [58], which is substantially less than that seen after several passages of *S. aureus* in subinhibitory concentrations of daptomycin alone [12, 16, 17]. The effect was the most pronounced in case of aminopenicillins, such as ampicillin or amoxicillin/clavulanic acid [58]. 

Interestingly, in an *in vitro* pharmacodynamic model of IE with simulated endocardial vegetations, the combination of daptomycin 6 mg/kg/day with cefepime 2 g bid was superior to daptomycin monotherapy in eradication of MRSA/VSSA but not of MRSA/hVISA [59]. Both strains were heterogeneously non-susceptible to daptomycin with the former having two-fold higher MIC than the latter.

In *in vitro* time-kill assays using clinical strains of* S. aureus *which developed non-susceptibility during treatment (daptomycin MIC 2–4 mg/L) and one *in vitro* selected non-susceptible strain (MIC 8 mg/L), treatment with a combination of daptomycin and oxacillin at 0.25 MIC showed modest increase of *in vitro* oxacillin bactericidal activity within 4–6 hours (1-2 log CFU/mL decrease in cell counts). An *in vivo* study of a rabbit model of aortic IE was then performed using initial daptomycin susceptible and non-susceptible clinical MRSA isolates. An increased bactericidal effect of the combination on the daptomycin non-susceptible *S. aureus* strains was established; it was more pronounced *in vivo* than *in vitro*. For the daptomycin-susceptible parental strains, the combination treatment with beta-lactams was not studied because daptomycin alone was found to be sufficiently bactericidal to cure the infection [56]. In spite of the observed “see-saw” effect (increased susceptibility to oxacillin) in daptomycin non-susceptible strains oxacillin monotherapy was not successful. All examined strains retained *mec*A gene.

### 5.3. Combinations of Daptomycin with Bacteriostatic Antibiotics

A somewhat unusual combination of daptomycin and TMP-SMX was studied in an *in vitro* pharmacodynamic model of IE with simulated endocardial vegetations. The TMP-SMX monotherapy was found inferior to the standard treatment of* S. aureus *IE [60]. The combination of daptomycin at 6 mg/kg/day and TMP-SMX at 160/800 mg bid was more rapidly bactericidal against daptomycin non-susceptible strains of* S. aureus *than daptomycin alone [59]. 

The efficacy of combination of clarithromycin and a beta-lactam or vancomycin for eradication of* S. aureus *biofilms has already been demonstrated [61]. A similar increase in bactericidal activity was observed in an *in vitro* model of biofilm using an MSSA strain. The combination of daptomycin 10 mg/kg/day and clarithromycin 250 mg bid was more effective in eradication of bacteria in the biofilm than daptomycin alone [53]. 

The mechanism behind the efficacy of some of the unusual combinations of antibiotics presented in this review is not clear. A concept of a mutant prevention concentration and mutant selection window hypothesis was proposed a decade ago [62]. Mutant prevention concentration is defined as the minimal concentration of antibiotic at which no single-step resistant mutants are recovered on agar plates when plated at high inoculum (>10^10^ cells) [62] while mutant selection window denotes a window between MIC_99_ and mutant prevention concentration in which resistant mutants are enriched (MIC_99_/mutant prevention concentration) [63]. It is thought to represent the MICs of susceptible and single-step resistant mutants. Both parameters are agent and bacterial-strain dependent. Mutant selection window is in some cases small with mutant prevention concentration well below the clinically achievable antibiotic concentrations, such as those of penicillin in case of MSSA. On the other hand, mutant selection window of rifampicin for MSSA is very broad (160,000) with mutant prevention concentration of 480 mg/L [63] which is far too high for clinical use. This may explain the high resistance observed in the laboratory. Combined treatment with two or more antibiotics may have a lower mutant prevention concentration, since two or more concomitant resistance mutations are necessary for growth; thus, reaching concentrations of antibiotics that exceed mutant prevention concentration is then feasible in a clinical setting [63]. Mutant selection window hypothesis has been confirmed for daptomycin *in vitro*, where the mutant selection window is about 2–5 with clinically achievable concentrations well above the mutant prevention concentration with a currently highest approved daily dose of 6 mg/kg [64, 65]. Nevertheless, with the high protein binding of daptomycin [4], the observed development of non-susceptibility* in vitro* and in clinical cases at a dose of 6 mg/kg/day suggests that the effective free drug concentrations are lower (as much as 90%) and that mutant prevention concentration is not reached. This speculation is further strengthened by the success of high doses of daptomycin (10 mg/kg/day), which are, even so, not approved by regulatory agencies for clinical use. A combination of daptomycin with other antibiotics may lower the mutant prevention concentration as has already been described for tobramycin-rifampicin combination [63].

## 6. Conclusions and Future Outlook

We have discussed combinations of daptomycin with other antibiotics, a new therapeutic strategy for treatment of complicated infections with *S. aureus*. Classical combination of daptomycin with rifampicin seems to be effective only for foreign-body infections while data for IE is somewhat contradictory. Combinations of daptomycin with beta-lactams, TMP-SMX, or clarithromycin provide a fresh strategy in combating infections with MDR* S. aureus*; the value of the new approaches is supported by the findings of *in vitro* and *in vivo* studies but is yet to be assessed and proven in clinical settings.

## Figures and Tables

**Table 1:** Data on previous vancomycin/daptomycin therapy in MRSA and MSSA clinical isolates non-susceptible to daptomycin.

Strain	Infection	Previous VAN therapy (days)	Reason for change	DAP therapy	MIC of DNS strain (days*)	Outcome	Ref.
MRSA	IE	Yes (No data)	Failure	Yes	MIC 2^ b ^ (No data)	No data	[26] (Case report)
MRSA	IE	Yes (47**)	Failure	Yes	MIC 4^a^(14)	Died (*C. albicans* septic shock)	[27] (Case report)
MRSA	IE	Yes (46***)	Failure	6 mg/kg q24 h	MIC 4^ a ^ (24)	Survived on alternative treatment	[30] (Case report)
MRSA	BSI	Yes (15)	Failure	6 mg/kg q24 h, then 8 mg/kg q24 h	MIC 4^ a ^ (6)	Died	[31] (Case report)
MRSA	BSI	Yes (No data)	No change	No	MIC 2^ a ^ (No data)	No data	[32] (Case series)
MRSA	UTI	Yes (12)	Failure	6 mg/kg q24 h	MIC 4^ a ^ (7)	Died	[29] (Case report)
MSSA	OM	Yes (60)	Failure	No	MIC 4^ a ^(No data)	Survived on alternative treatment(nafcillin +rifampin)	[34] (Case report)

DAP: daptomycin; VAN: vancomycin; DNS: daptomycin non-susceptible; UTI: urinary tract infection; BSI: bloodstream infection; IE: infective endocarditis; OM: osteomyelitis. MRSA: meticillin-resistant *S. aureus*; MSSA: meticillin-susceptible *S. aureus*; *duration (days) of treatment with daptomycin prior to isolate was obtained; **vancomycin for 12 days, then teicoplanin 35 days; ***2 courses: 6 weeks initially, additional 4 days after relapse; (a) broth microdilution (b) *E*test. All initial isolates were susceptible to daptomycin; MIC: minimal inhibitory concentration (mg/L).

**Table 2:** Information on *in vivo* synergy of antibiotic combinations.

Model	Strain	Combination	Observation	Ref.
Experimental model of IE	MRSA	Daptomycin 6 mg/kg q24 h + rifampin 300 mg q8 h	Rifampin and gentamicin antagonized/delayed the bactericidal activity of daptomycin	[39]
		Daptomycin 6 mg/kg q24 h + gentamicin 1.3 mg/kg q12 h		

Rabbit model of IE	MRSA	Daptomycin 6 mg/kg q24 h + gentamicin 1 mg/kg q8 h	60% vegetations sterilized	[41]
		Daptomycin 6 mg/kg q24 h + rifampin 300 mg q8 h	20% vegetations sterilized	

Rabbit model of IE	DNS MRSA	Daptomycin 12 mg/kg q24 h + oxacillin 200 mg/kg q8 h	Enhanced bacterial clearance from tissues	[56]

Case report on IE	MRSA with progressive loss of susceptibility during treatment	Treated with vancomycin, then daptomycin 6 mg/kg q24 h, then daptomycin 12 mg/kg q24 h + rifampin 300 mg q8 h	Clinical success	[42]

Rabbit acute osteomyelitis model	MRSA	Daptomycin 6 mg/kg q24 h	Failure to eradicate bacteria	[50]
		Daptomycin 6 mg/kg q24 h + rifampin 20^a ^ mg/kg q12 h	Eradication of bacteria: bone 100%, bone marrow 89%, and joint fluid 44%	

Guinea pig foreign-body infection model	MRSA	Daptomycin 20 mg/kg q24 h + rifampin 12.5 mg/kg q12 h	25% cure	[51]
		Daptomycin 30^b^ mg/kg q24 h + rifampin 12.5 mg/kg q12 h	67% cure	
		Vancomycin 15 mg/kg q12 h + rifampin 12.5 mg/kg q12 h	8% cure	

Rat foreign-body infection model	MRSA	Daptomycin 100^c^ mg/kg q24 h + rifampin 25 mg/kg q12 h	94% cure	[53]
		Daptomycin 45 mg/kg q24 h + rifampin 25 mg/kg q12 h	64% cure	
		Vancomycin 50 mg/kg q12 h +rifampin 25 mg/kg q12 h	25% cure	

In vitro PK/PD model of SEV	DNS MRSA	Daptomycin 6 mg/kg q24 h + TMP/SMX 160/800 mg q12 h	Bactericidal activity reached at 8 hours	[59]
		Daptomycin 6 mg/kg q24 h + cefepime 2 g q8 h		

In vitro PK/PD model of SEV	MSSA, MRSA	Daptomycin 6–8 mg/kg q24 h + gentamicin 5 mg/kg as a single dose; or + gentamicin 1 mg/kg q8 h, three doses only	Daptomycin 6–8 mg/kg/day combined with one 5mg/kg dose of gentamicin was bactericidal in 4 h	[66]

In vitro PK/PD model of SEV	GISA, MRSA	Daptomycin 3–6 mg/kg q24 h + arbekacin 100 mg q12 h	Synergy, but regrowth in 48 h in the regimen with daptomycin 4 mg/kg/day	[67]

Equivalent dosing to (a) rifampin 10 mg/kg q12 h oral administration (b) daptomycin 6 mg/kg q24 h (c) daptomycin 10 mg/kg q24 h in humans; PK/PD: pharmacokinetic/pharmacodynamic; SEV: simulated endocardial vegetations; TMP/SMX: trimethoprim-sulfamethoxazole; IE: infective endocarditis, DNS: daptomycin non-susceptible; MRSA: meticillin-resistant *S. aureus*; GISA: glycopeptide-intermediate *S. aureus.*

**Table 3:** *In vitro* synergy of antibiotic combinations.

Method	Strain	Combination	Observation	Ref.
*E*test and time-kill study	hGISA/MSSA	Daptomycin with vancomycin, gentamicin, rifampin, linezolid, quinupristin/ dalfopristin, ampicilin-sulbactam	*E*test: additive effect with daptomycin + vancomycin and daptomycin + gentamycin, other combinations with daptomycin indifferent Time-kill study: additive effect with daptomycin + gentamycin, other combinations with daptomycin indifferent	[40]

*E*test and time-kill study	GISA/MRSA	Daptomycin with vancomycin, gentamicin, rifampin, linezolid, quinopristine/ dalfopristine, ampicilin-sulbactam	*E*test: additive effect only with daptomycin + gentamycin, other combinations with daptomycin indifferent Time-kill study: indifference	[40]

Time-kill study	MRSA	Daptomycin + gentamicin	Synergy in all three strains	[41]

Time-kill study	MRSA	Daptomycin + rifampin	Antagonism in one strain and indifference in two other strains	[41]

Time-kill study	MRSA	Daptomycin at 0.0625 MIC to 2 MIC + oxacillin or ampicillin- sulbactam	Synergy of daptomycin at 0.5 MIC + oxacillin 32 mg/L or ampicillin-sulbactam 2–8 mg/L (ampicillin)	[57]

*In vitro* model of biofilm	MSSA	Daptomycin 10 mg/kg/d + clarithromycin 250 mg q12 h	Sustained bactericidal activity against planctonic and biofilm bacteria	[54]

*In vitro* model of biofilm	MRSA	Daptomycin 10 mg/kg/d + rifampin at 600 mg q24 h	Sustained bactericidal activity against planctonic and biofilm bacteria	[54]

*In vitro* selection for resistance	MRSA and GISA	Daptomycin + 0.25 MIC ampicillin, amoxicillin-clavulanic acid, gentamicin, rifampin	Combination with ampicillin, amoxicillin-clavulanic acid delayed/prevented occurrence of non-susceptibility; rifampin delayed non-susceptibility	[58]

Checkerboard method	MRSA, MSSA	Daptomycin + rifampicin, moxifloxacin or fusidic acid	Daptomycin + fusidic acid: antagonism in one MSSA strain reported	[68]

MRSA: meticillin-resistant *S. aureus*; MSSA: meticillin-susceptible *S. aureus;* GISA: glycopeptide-intermediate *S. aureus*; hGISA: heteroresistant glycopeptide-intermediate *S. aureus; *MIC: minimal inhibitory concentration (mg/L).
